# Interaction of TWEAK with Fn14 leads to the progression of fibrotic liver disease by directly modulating hepatic stellate cell proliferation‡

**DOI:** 10.1002/path.4707

**Published:** 2016-03-29

**Authors:** Annika Wilhelm, Emma L Shepherd, Aldo Amatucci, Mamoona Munir, Gary Reynolds, Elizabeth Humphreys, Yazid Resheq, David H Adams, Stefan Hübscher, Linda C Burkly, Christopher J Weston, Simon C Afford

**Affiliations:** ^1^ Centre for Liver Research and National Institute for Health Research (NIHR) Birmingham Liver Biomedical Research UnitUniversity of BirminghamUK; ^2^ Department of ImmunologyBiogenCambridgeMAUSA; ^3^ Medizinische Klinik 5/Department of Internal Medicine 5Universitätsklinikum Erlangen/University Medical Centre ErlangenGermany; ^4^ Department of Cellular PathologyUniversity Hospital Birmingham NHS Foundation TrustBirminghamUK

**Keywords:** TNF family, liver fibrosis, myofibroblast

## Abstract

Tumour necrosis factor‐like weak inducer of apoptosis (TWEAK) and its receptor fibroblast growth factor‐inducible 14 (Fn14) have been associated with liver regeneration in vivo. To further investigate the role of this pathway we examined their expression in human fibrotic liver disease and the effect of pathway deficiency in a murine model of liver fibrosis. The expression of Fn14 and TWEAK in normal and diseased human and mouse liver tissue and primary human hepatic stellate cells (HSCs) were investigated by qPCR, western blotting and immunohistochemistry. In addition, the levels of Fn14 in HSCs following pro‐fibrogenic and pro‐inflammatory stimuli were assessed and the effects of exogenous TWEAK on HSCs proliferation and activation were studied in vitro. Carbon tetrachloride (CCl_4_) was used to induce acute and chronic liver injury in TWEAK KO mice. Elevated expression of both Fn14 and TWEAK were detected in acute and chronic human liver injury, and co‐localized with markers of activated HSCs. Fn14 levels were low in quiescent HSCs but were significantly induced in activated HSCs, which could be further enhanced with the profibrogenic cytokine TGFβ in vitro. Stimulation with recombinant TWEAK induced proliferation but not further HSCs activation. Fn14 gene expression was also significantly up‐regulated in CCl_4_ models of hepatic injury whereas TWEAK KO mice showed reduced levels of liver fibrosis following chronic CCl_4_ injury. In conclusion, TWEAK/Fn14 interaction leads to the progression of fibrotic liver disease via direct modulation of HSCs proliferation, making it a potential therapeutic target for liver fibrosis. © 2016 The Authors. *The Journal of Pathology* published by John Wiley & Sons Ltd on behalf of Pathological Society of Great Britain and Ireland.

## Introduction

Liver fibrosis occurs as a result of excessive accumulation of extracellular matrix (ECM) and is a common consequence of the majority of chronic liver diseases. It can progress to cirrhosis, a major cause of liver‐related morbidity and mortality, and in a small but significant number of cases leads to the development of liver cancer 1. Liver myofibroblasts are considered to be a key regulator of liver fibrogenesis, due to their ability to produce ECM, proliferate and migrate 2. They are not present in healthy livers but accumulate after injury and are thought to derive primarily from hepatic stellate cells (HSCs) 3, 4. Gaining a greater understanding of liver fibrogenesis could aid in the development of new treatment options for preventing progression to end‐stage cirrhosis.

The TNF family member TNFlike weak inducer of apoptosis (TWEAK; *TNFSF12*) is expressed as a type II transmembrane protein that can be cleaved proteolytically to generate a soluble protein 5. *In vitro* data have shown that TWEAK may be involved in several cellular processes, including proliferation, differentiation and migration 6. TWEAK signals through its receptor, fibroblast growth factor‐inducible 14 (Fn14; *TNFRSF12A*). Interactions between TWEAK and Fn14 have been reported to modulate fibrosis in several organs, including the heart, kidney, colon and muscle 7.

In the liver, the signalling and functional responses of TWEAK and Fn14 have mainly been investigated in association with liver regeneration *in vivo* and only to a limited extent with fibrosis. Fn14 expression is rapidly up‐regulated following liver injury 8, 9, 10 and is associated with liver progenitor cells (LPCs) 9, 11, 12, whereas TWEAK expression has mainly been detected in liver‐derived natural killer cells and macrophages 9. LPCs normally expand as a mechanism for liver regeneration following severe acute and chronic liver injury, to help replace damaged hepatocytes and biliary epithelial cells 13. *In vivo* studies have demonstrated that Fn14 KO mice treated with the choline‐deficient, ethionine‐supplemented (CDE) diet to induce LPC proliferation had fewer LPCs compared with their wild‐type (WT) controls 9. In addition, those mice also exhibited a decreased fibrogenic response, including less deposition of collagen 9. Similarly, Fn14 KO mice that had subacute liver injury and were fed the high‐fat Lieber deCarli diet supplemented with 2% alcohol displayed defective liver wound‐healing responses, including fewer LPCs and myofibroblasts and less deposition of collagen 14. LPCs are thought to be involved in the expansion of ductular reactive cells, and evidence has shown that a correlation exists between the presence of ductular reactions and the severity of fibrosis 15, supporting a circumstantial link between TWEAK‐mediated LPC proliferation and fibrogenesis 9, 16.

Thus far, it has not been determined whether TWEAK has effects on myofibroblasts and HSCs. However, the current findings suggest that TWEAK–Fn14 interaction might promote liver fibrogenesis; but whether its mode of action is via HSCs or whether it is through crosstalk with LPCs is currently unknown. In addition, the expression and function of TWEAK and Fn14 has not been investigated extensively in human fibrotic liver disease.

The aims of our study were to: (a) investigate the expression and localization of TWEAK and Fn14 in human liver tissue from normal healthy donor organs and compare with liver tissue taken from patients with established end‐stage disease; (b) determine the expressions and roles of TWEAK and Fn14 in isolated human HSCs *in vitro*; and (c) evaluate the role of TWEAK in a carbon tetrachloride (CCl_4_) model of acute and chronic liver injury.

## Materials and methods

### Human liver tissue

Human liver tissue was obtained with ethical approval from the liver transplant unit [Queen Elizabeth Hospital (QEH), Birmingham, UK]. Explanted liver specimens were obtained from consenting patients with non‐alcoholic steatohepatitis (NASH), alcoholic liver disease (ALD), primary biliary cirrhosis (PBC), primary sclerosing cholangitis (PSC), autoimmune hepatitis (AIH) and acute liver failure (ALF). Normal donor livers surplus to surgical requirements, or as a by‐product of surgical resection, served as normal controls (NL). All patients gave informed written consent and studies were approved by the Local Research Ethics Committee (Reference No. 06/Q2702/61).

### Mice

Male 6–8 week‐old homozygous *TWEAK* KO mice 17, along with their WT controls (Balb/c background), were given CCl_4_ via gavage (Sigma‐Aldrich; 1 ml/kg dissolved in mineral oil) to induce acute and chronic liver injury. Mineral oil alone was used as a control. In the acute models, the mice received one dose of CCl_4_ and were sacrificed 24, 48, 72 or 96 h after administration. In the chronic model, the mice were administered CCl_4_ once weekly for each of 4 weeks and sacrificed 3 days after the last dose. All animal procedures were conducted in accordance with Cambridge (MA, USA) laws and the Institutional Animal Care and Use Committee, UK (Protocol No. 0458–2013).

### Isolation of primary human HSCs

Primary human HSCs were isolated and grown as described previously 18. Uninvolved liver tissue from patients undergoing surgical resection for liver malignancy was dissociated by pronase/collagenase digestion and HSCs separated by buoyancy centrifugation. HSCs were cultured on plastic dishes, on which cells were automatically activated. HSC cultures were in some instances stimulated with optimum concentrations of recombinant bFGF (0.61 nm), TGFβ1 (0.40 nm), TNFα (0.57 nm), IFNγ (5.95 nm; PeproTech, London, UK), or for gene expression studies HSCs were incubated with TWEAK (0.06–29.4 nm; Biogen, USA) for 24 h.

### Light microscopy and immunostaining

Paraffin‐embedded mouse liver tissue cut to 3 µm thickness was stained with haematoxylin and eosin (H&E), using standard protocols. For immunohistochemical studies, snap‐frozen human sections cut at 5–7 µm thickness were incubated with mouse anti‐Fn14 (3.7 µg/ml; mP4A8) or mouse anti‐TWEAK (3.24 µg/ml; mP2D10; both Biogen) diluted in TBS/0.1% Tween 20 for 1 h. Staining was detected with immPRESS anti‐mouse secondary antibody and 3,3′‐diaminobenzidine (both Vector Laboratories, Peterborough, UK). The sections were then counterstained with haematoxylin (VWR). Negative controls were performed using matched isotype control antibodies, which uniformly demonstrated no reaction.

For immunofluorescent staining, HSCs were grown in Ibidi Slide 12 chamber slides and fixed in methanol. Human sections or fixed HSCs were dual‐stained with anti‐Fn14 (mP4A8), anti‐TWEAK (mP2D10), anti‐α‐smooth muscle actin (α‐SMA; 1:50; 1A4; Dako) anti‐vimentin (1:100; V9; eBioscience, Hatfield, UK) or anti‐CK19 (1:200; Ks 19.1; Progen Biotechnik, Heidelberg, Germany) and fluorophore‐labelled secondary antibodies (AlexaFluor 488 anti‐mouse and AlexaFluor 546/594 anti‐mouse, both 1:500; Life Technologies). In some instances, sequential staining was performed as previously described 19. DAPI was used as a nuclear counterstain. The slides were visualized using a Zeiss LSM 510 confocal microscope (Carl Zeiss, Germany).

### Quantification of α‐SMA ^+^ and Sox9^+^ cells and collagen

For α‐SMA and Sox9 quantification, paraffin‐embedded mouse liver sections were stained on an automated immunostainer (Dako), using a mouse on mouse ImmPRESS HRP kit (Vector Laboratories) according to the manufacturer's instructions, using anti‐mouse α‐SMA (1:200; ASM‐1; Vector Laboratories) or anti‐rabbit Sox9 (1:500; Millipore) with a Vector ImmPRESS horseradish peroxidase rabbit secondary antibody. Ten non‐overlapping fields (×20 objective) from each section were captured using a light microscope (Carl Zeiss) with identical illumination and exposure. Digital image analysis was performed using ImageJ software for α‐SMA quantification. Sox9^+^ cells were counted manually. For collagen quantification, paraffin‐embedded mouse liver sections or snap‐frozen human liver sections were stained with Sirius red, using a standard protocol. Collagen values were expressed as the percentage of the total area of a section occupied by Sirius red staining.

### Cell proliferation assay

HSCs were stimulated with recombinant TWEAK (5.88–58.8 nm; Biogen), or PDGF‐BB (0.41 nm; Miltenyi Biotec, Surrey, UK) for 48 h. A commercially available CyQUANT NF Proliferation Assay Kit (Life Technologies) was used, according to the manufacturer's instructions, to evaluate cell proliferation. Fluorescence was measured at 480/520 nm on a Synergy HT plate reader (Bio‐Tek). Background fluorescence was subtracted and data were expressed in arbitrary units of fluorescent intensity compared to an unstimulated control. Further methods are described in supplementary materials and methods online.

### Statistical analysis

Results are presented as median with interquartile range (IQR). Statistical analysis was performed using the Kruskall–Wallis test, Mann–Whitney U‐test or Spearman correlation co‐efficient, using Prism software (GraphPad, USA); *p* < 0.05 was considered statistically significant.

## Results

### TWEAK and Fn14 are up‐regulated in fatty and immune‐mediated liver diseases and in an experimental model of acute and chronic liver injury

To determine the roles of TWEAK and Fn14 in liver disease, we first assessed the expression levels of TWEAK and Fn14 in a variety of human end‐stage liver diseases and ALF. We used liver tissue taken from patients with ALD and NASH as examples of fatty liver diseases, as they are associated with steatosis in the liver, which is largely driven by lifestyle and environmental factors. We also included liver tissue sampled from patients with end‐stage PBC, PSC and AIH, which were classed as immune‐mediated liver diseases, where the immunological response might modulate expression levels of TWEAK and Fn14.

**Figure 1:**
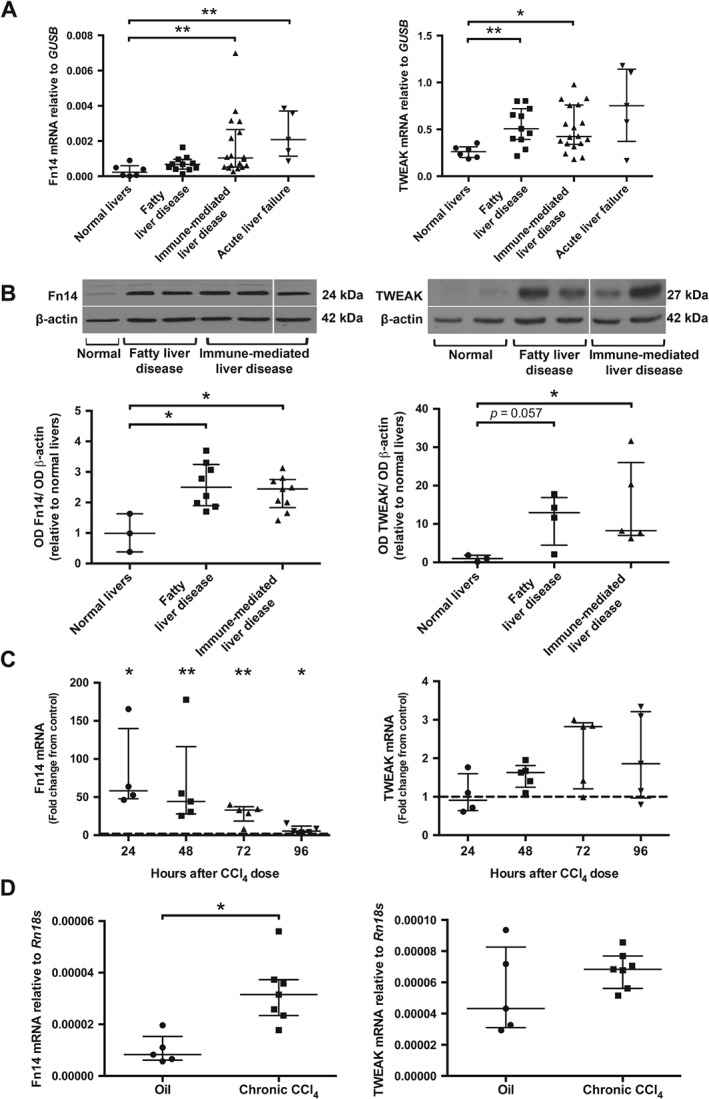
TWEAK and Fn14 expression in normal, chronic and acute liver disease in humans and mice. (A) Expression of Fn14 (TNFRSF12A) and TWEAK (TNFSF12) mRNA was assessed by qPCR in normal livers (n = 6), chronic fatty liver disease (n = 11; ALD n = 5 and NASH n = 6), chronic immune‐mediated liver disease (n = 18; PBC n = 6, PSC n = 6 and AIH n = 6) and ALF (n = 5). Gene expression is shown relative to GUSB, using the 2^–ΔCt^ method. (B) Representative western blot image of Fn14, TWEAK and β‐actin levels in normal livers, chronic fatty liver diseases and chronic immune‐mediated liver diseases: densitometry analysis of Fn14 protein expressed in normal donor livers (n = 3), fatty liver disease (n = 8; ALD n = 5 and NASH n = 3) and immune‐mediated liver disease (n = 9; PBC n = 3, PSC n = 3 and AIH n = 3) normalized to β‐actin expression; densitometry analysis of TWEAK protein expressed in normal donor livers (n = 3), fatty liver disease (n = 4; ALD n = 1 and NASH n = 3) and immune‐mediated liver disease (n = 5; PBC n = 3, PSC n = 1 and AIH n = 1) normalized to β‐actin expression. (C, D) Murine Fn14 (Tnfrsf12a) and TWEAK (Tnfsf12) mRNA were analysed in liver tissue from WT animals by qPCR following (C) acute or (D) chronic CCl_4_‐induced liver injury, or mineral oil control, at the time points indicated; gene expression is shown relative to Rn18s, using the 2^–ΔCt^ method: dashed line, mRNA levels of mineral oil control‐treated KO and WT mice; each dot represents one independent sample; data are shown as median with IQR; statistical significance is represented as *p < 0.05 or **p < 0.01 (Mann–Whitney U‐test)

The levels of *Fn14* mRNA (*TNFRSF12A*) were significantly increased in immune‐mediated liver diseases (5.26‐fold; *p <* 0.01) and ALF (7.39‐fold; *p <* 0.01) when compared to normal human livers (Figure 1A). A modest increase was observed in fatty liver diseases, although this did not reach significance (2.19‐fold; *p* = 0.06). Importantly, Fn14 protein levels in fatty and immune‐mediated liver diseases were both elevated compared to normal donor tissue (Figure 1B). A significant increase of *TWEAK* mRNA (*TNFSF13*) was observed in fatty livers (2.05‐fold; *p <* 0.01) and immune‐mediated liver diseases (1.93‐fold; *p <* 0.05), whereas *TWEAK* mRNA levels in ALF were not significantly different (2.89‐fold; *p* = 0.13) compared to normal human livers (Figure 1A). TWEAK protein levels were significantly increased in immune‐mediated liver diseases (8.49‐fold; *p <* 0.05) and a trend towards an increase was detected in fatty livers (13.31‐fold; *p* = 0.057).

The extent of fibrosis in matched tissue sections was assessed by Sirius red staining as a surrogate marker of disease progression. The mRNA and protein levels of both Fn14 and TWEAK positively correlated with the extent of liver fibrosis, and therefore the extent of hepatic injury (Supplementary Figure S1).

To further investigate the expression of TWEAK and Fn14 during liver injury, WT mice were challenged with CCl_4_ orally to induce toxic hepatic damage. Analysis of murine livers following acute hepatic injury revealed a significant increase in *Fn14* mRNA (*Tnfrsf12a*) (58‐, 44‐, 33‐ and six‐fold changes at 24, 48, 72 and 96 h post‐CCl_4_ challenge, respectively) (Figure 1C). In contrast, levels of *TWEAK* mRNA did not differ significantly between experimental groups. A similar effect was observed for a chronic CCl_4_‐induced model of liver fibrosis, where *Fn14* mRNA was up‐regulated 3.18‐fold, (*p <* 0.01), whereas *TWEAK* mRNA was not elevated above baseline (Figure 1D).

### TWEAK and Fn14 are expressed by myofibroblasts in fibrotic septa

**Figure 2:**
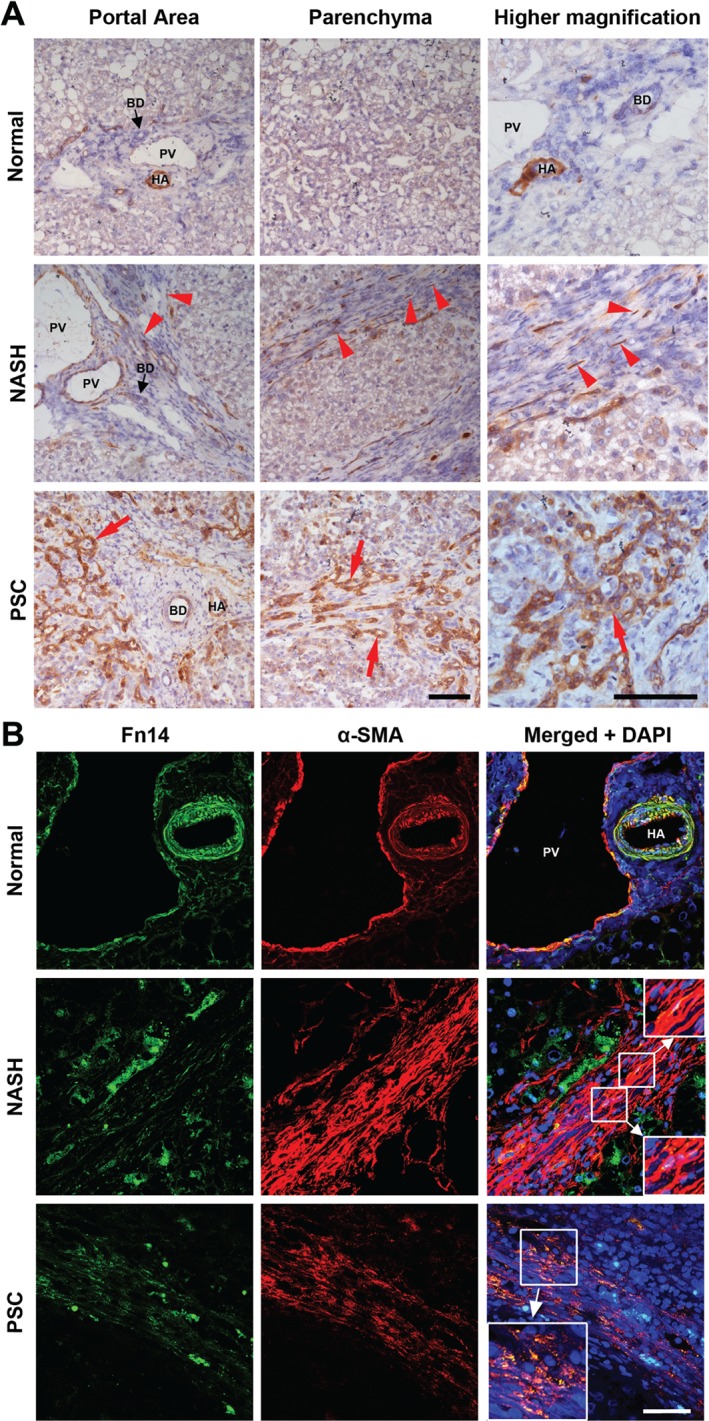
Fn14 is expressed by myofibroblasts in human liver disease. (A) Representative immunostaining of Fn14 (brown) in human liver samples from normal donors and patients with chronic end‐stage NASH or PSC; red arrowheads, spindle‐shaped cells in the fibrotic scar; red arrows, ductules in foci of ductular reaction; scale bars = 100 µm. (B) Representative confocal images showing localization of Fn14 (green) expressing cells relative to those expressing the myofibroblast/activated HSCs marker α‐SMA in tissue samples from normal donors and patients with NASH or PSC: co‐localization of Fn14 and α‐SMA resulted in pseudocolour yellow; (inset) digitally enlarged image; blue, DAPI used as a nuclear counterstain; scale bar = 50 µm; PV, portal vein; HA, hepatic artery; BD, bile duct

To determine the localization of Fn14 in human liver diseases, histological samples from NL, NASH and PSC were stained by immunohistochemistry (Figure 2A). In normal human liver tissue, Fn14 expression was low in the portal area and no staining was detected in the parenchyma. Higher‐power imaging of the portal areas localized Fn14 to blood vessels, in particular smooth muscle cells in the arterial wall, and bile ducts. Fn14 staining was also present in blood vessels in tissue sections taken from patients with NASH, and in myofibroblast‐like cells within the fibrotic septa (Figure 2A). Immunofluorescent dual staining of tissue sections confirmed that Fn14 expression (green) co‐localized with α‐SMA (red), a common marker for myofibroblasts and smooth muscle cells, in normal livers and in tissue from patients with NASH and PSC (Figure 2B). Samples from patients with NASH and PSC also demonstrated co‐localization of Fn14 (green) and CK19 (red), a biliary marker that highlights bile ducts and areas of ductular reactivity (Supplementary Figure S2).

**Figure 3:**
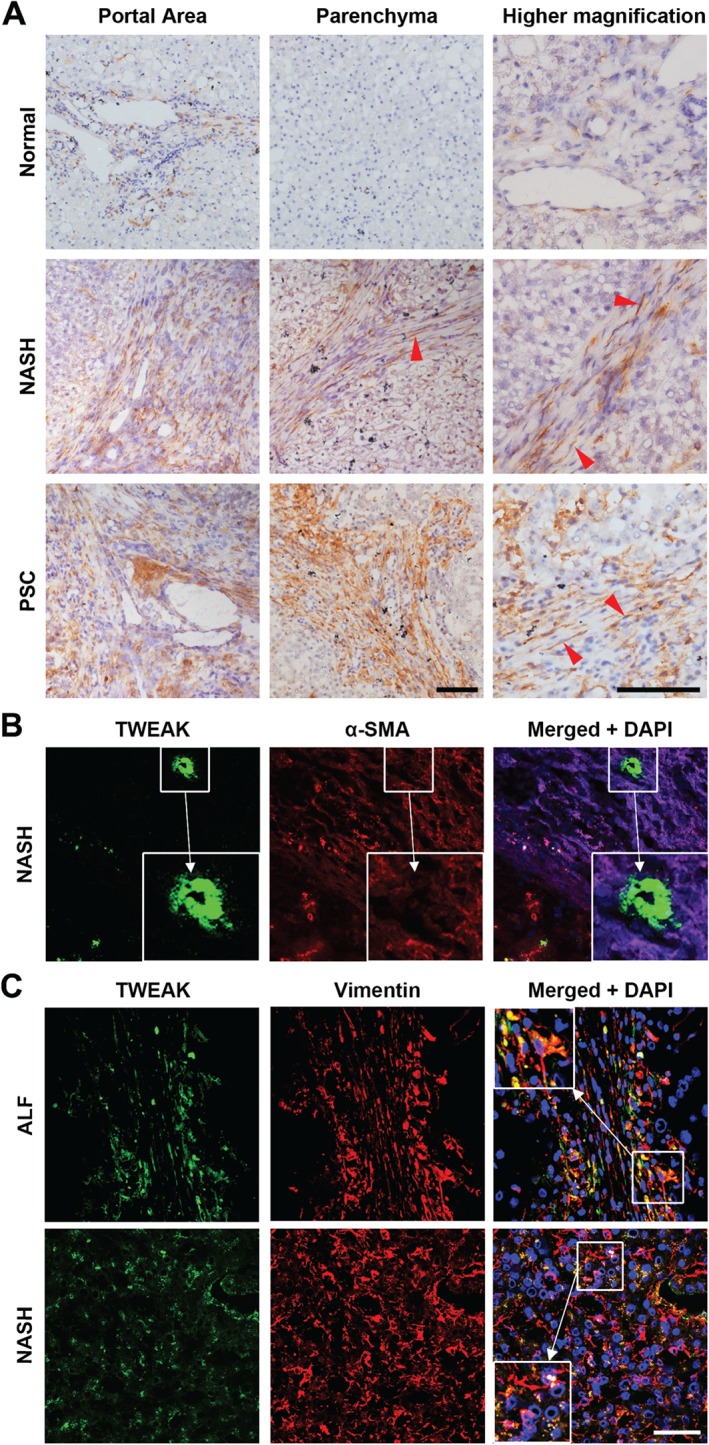
Expression of TWEAK by stromal cells in diseased human liver tissue. (A) Representative immunostaining of TWEAK (brown) in human liver samples from normal donors and patients with NASH or PSC; red arrowheads, spindle‐shaped cells in the fibrotic scar; scale bars = 100 µm. (B) Representative confocal images showing association of TWEAK expressing cells (green) with those expressing the fibroblast marker α‐SMA (red) in tissue samples from patients with NASH and (C) vimentin (red) in tissue samples from patients with ALF and NASH; co‐localization of TWEAK and α‐SMA/vimentin resulted in pseudocolour yellow; (inset) digitally enlarged image; blue, DAPI used as a nuclear counterstain; scale bar = 50 µm

Expression of TWEAK in normal liver tissue was weak and limited to the portal region (Figure 3A). Increased levels of TWEAK were detected in tissue sections from NASH and PSC patients and localized to the portal area and fibrotic septa. TWEAK was associated with spindle‐shaped cells consistent with a myofibroblast phenotype, similar to that seen for Fn14 in NASH and PSC, and was also detected in the sinusoids (Figure 3A). To further investigate the identity of these cells, dual‐colour immunofluorescent staining for TWEAK (green) and α‐SMA (red) was performed in tissue samples from patients with NASH (Figure 3B), in addition to detection of the HSC and myofibroblast marker, vimentin (red) and TWEAK (green), in NASH and ALF samples (Figure 3C). Co‐localization of TWEAK with α‐SMA and vimentin confirmed the expression of TWEAK by hepatic myofibroblasts; it also showed co‐localization of TWEAK with HSCs/myofibroblasts in the sinusoids in NASH livers (Figure 3C).

### Hepatic stellate cells express Fn14 in vitro and proliferate in response to TWEAK stimulation

**Figure 4:**
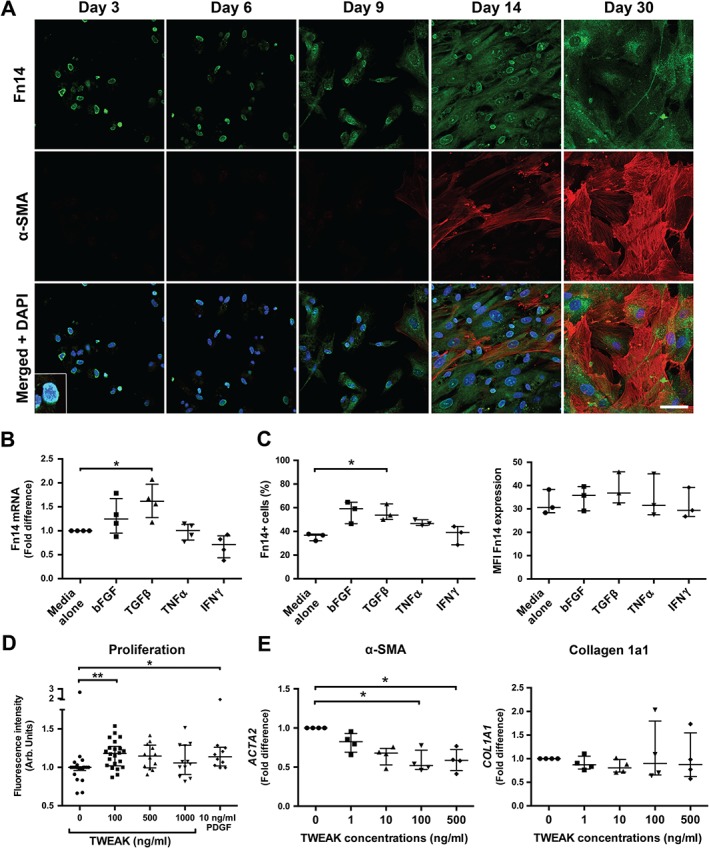
HSCs up‐regulate Fn14 expression during activation and proliferate in response to TWEAK stimulation in vitro. HSCs were isolated from normal liver tissue and plated on plastic to initiate activation. (A) Confocal images of Fn14 (green) and α‐SMA (red) expression in HSCs during a 30‐day activation period; (inset) digitally enlarged image; blue, DAPI used as a nuclear counterstain; scale bar = 50 µm. (B, C) Fn14 expression in HSCs following treatment with bFGF, TGFβ1, TNFα or IFNγ for 24 h in vitro was analysed by (B) qPCR, with gene expression shown as fold change relative to GUSB (n = 4 HSCs isolates), and (C) flow cytometry, expressed as the fraction of Fn14^+^ cells, and expression level, as determined by median fluorescent intensity (MFI) (n = 3); HSCs in medium alone served as control; each dot represents cells from one independent HSCs isolation. (D) HSCs were stimulated with TWEAK (100–1000 ng/ml) or PDGF‐BB (10 ng/ml) for 48 h in vitro and cell numbers were quantified using the CyQuant assay to determine cell proliferation (n = 4–7); each dot represents replicates from independent HSCs isolations; data are expressed as fold change above untreated control. (E) HSCs were stimulated with increasing concentrations of TWEAK for 24 h and expression of ACTA2 and COL1A1 was measured by qPCR; gene expression is shown relative to GUSB, using the 2^–ΔCt^ method (n = 4); each dot represents cells from one independent HSC isolation; data are shown as median with IQR; statistical significance is represented as *p < 0.05 or **p < 0.01 (Kruskal–Wallis test)

Imaging studies identified the presence of Fn14^+^ cells in the fibrous septa of diseased liver tissue which had the morphological appearance of myofibroblasts. Therefore, we wanted to investigate whether HSCs, the progenitors of myofibroblasts, also expressed Fn14 and how this process may be regulated. Quiescent HSCs were isolated from healthy donor liver tissue and plated on plastic to induce activation of the cells over a period of 14 days. Expression of Fn14 (green) by HSCs was detected by immunofluorescence by day 3 post‐isolation, and after 14 days the cells expressed α‐SMA (red), confirming differentiation into activated HSCs (Figure 4A). At days 3–6, Fn14 had a perinuclear distribution that became more diffuse and cytoplasmic with prolonged culture. We then investigated factors required for the regulation of Fn14 expression. *Fn14* mRNA was increased in activated HSCs following stimulation with TGFβ1 for 24 h, whereas bFGF, TNFα and IFNγ had little effect (Figure 4B). Flow‐cytometry analysis revealed that approximately 90% of activated HSCs had intracellular stores of Fn14 (data not shown). On average, 36% (±3%) of HSCs expressed Fn14 on the cell surface (Figure 4C). This was significantly increased with stimulation by the pro‐fibrogenic cytokine TGFβ1 (56 ± 6.8%; *p <* 0.05) and, although bFGF enhanced the proportion of cells expressing Fn14, this did not achieve significance (57% ± 9.3%). Treatment with TNFα (47% ± 2.5%) or IFNγ (37% ± 7.9%) had no effect on Fn14 cell surface expression by HSCs. This effect was not associated with an increase in the extent of surface expression/Fn14^+^ cell, as determined by the median fluorescent intensity (MFI) (Figure 4C).

We then investigated whether TWEAK could modulate HSC function. First, we measured endogenous secreted TWEAK in HSC supernatant to determine whether these cells contribute to levels of soluble (s)TWEAK present in the circulation. We found a median sTWEAK concentration of 25 pg/ml/30 000 cells in culture medium, confirming that HSCs are a source of this protein *in vitro*. To determine whether increased levels of sTWEAK were able to elicit responses in HSCs, we treated cultures of primary human HSCs with 100–1000 ng/ml recombinant human TWEAK; mimicking extensive changes in local concentrations of TWEAK that might be expected in disease liver tissue. Consistent with this, treatment of HSCs with 100 ng/ml TWEAK significantly enhanced the proliferative potential of HSCs following 48 h treatment (*p <* 0.01), a response that was similar to that observed for the known mitogen PDGF‐BB (10 ng/ml). Higher concentrations of TWEAK did not significantly increase HSCs proliferation, but equally did not lead to a reduction in cell number, as might be expected from TWEAK‐induced cell death (Figure 4D). We then assessed HSC activation following treatment with TWEAK by measuring the levels of α‐SMA (*ACTA2*) and collagen 1a1 (*COL1A1*) mRNA. The expression of *α‐SMA* mRNA was reduced following TWEAK exposure at 100 and 500 ng/ml compared to untreated HSCs (*p <* 0.05), while levels of *COL1A1* were unchanged following TWEAK stimulation at all concentrations tested (Figure 4E).

### TWEAK is critical for liver fibrogenesis following acute hepatic injury in vivo

**Figure 5:**
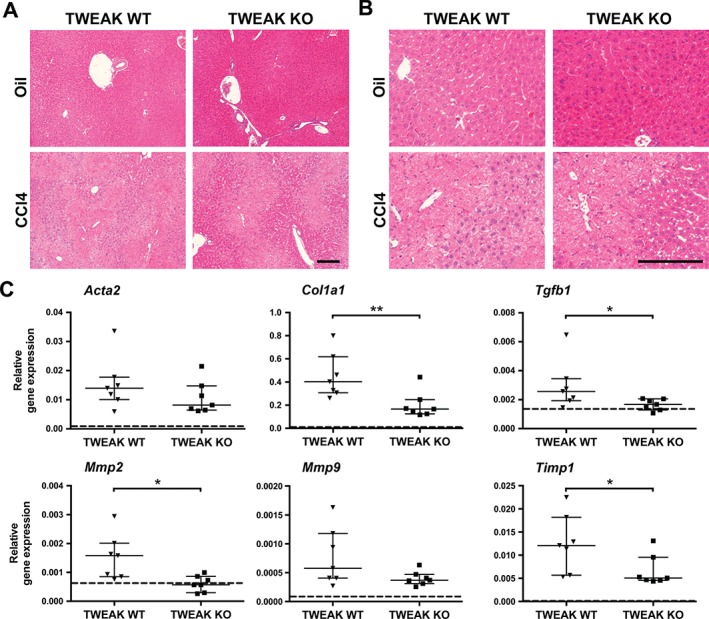
TWEAK KO animals show reduced levels of fibrotic mediators following CCl_4_‐induced acute liver injury; TWEAK KO and corresponding WT mice were injected with a single dose of CCl_4_ and killed 72 h afterwards. (A) Representative images of H&E staining from TWEAK KO and WT mice. (B) Digitally enlarged images of H&E staining from TWEAK KO and WT mice. (C) Expression levels of fibrogenic mediators were analysed by qPCR in CCl_4_‐treated TWEAK KO mice and relevant controls (n = 5–7); gene expression is shown relative to Gapdh, using the 2^–ΔCt^ method; Data are shown as median with IQR; each dot represents one mouse; statistical significance is represented as *p < 0.05 or **p < 0.01 (Mann–Whitney U‐test); dashed line, mRNA levels of mineral oil control‐treated KO and WT mice; scale bar = 200 µm

To further dissect the functional role of TWEAK in hepatic injury and fibrogenesis, TWEAK KO mice, along with their matched WT controls, were subjected to a single CCl_4_ dose and subsequently killed after 72 h. Following CCl_4_ administration, TWEAK KO mice had similar levels of liver injury compared to their WT controls, as histological examination demonstrated foci of centrilobular necrosis and inflammation in both KO and WT mice treated with CCl_4_ (Figure 5A). Liver tissue from TWEAK KO mice showed significantly lower levels of *Col1a1* (*p <* 0.01) and *Tgfb1*, which is a key pro‐fibrogenic growth factor that promotes HSCs activation (*p <* 0.05) (Figure 5B). In addition, mRNA levels for enzymes involved in matrix remodelling, including *Mmp2* (*p <* 0.05) and tissue inhibitor of metalloproteinase‐1 (*Timp1*; *p <* 0.05) were reduced in TWEAK KO mice compared to their WT controls. The levels of *Acta2* (*p =* 0.31) and *Mmp9* (*p =* 0.14) mRNA remained unchanged.

### TWEAK KO mice present with reduced liver fibrosis upon chronic CCl_4_‐treatment

**Figure 6:**
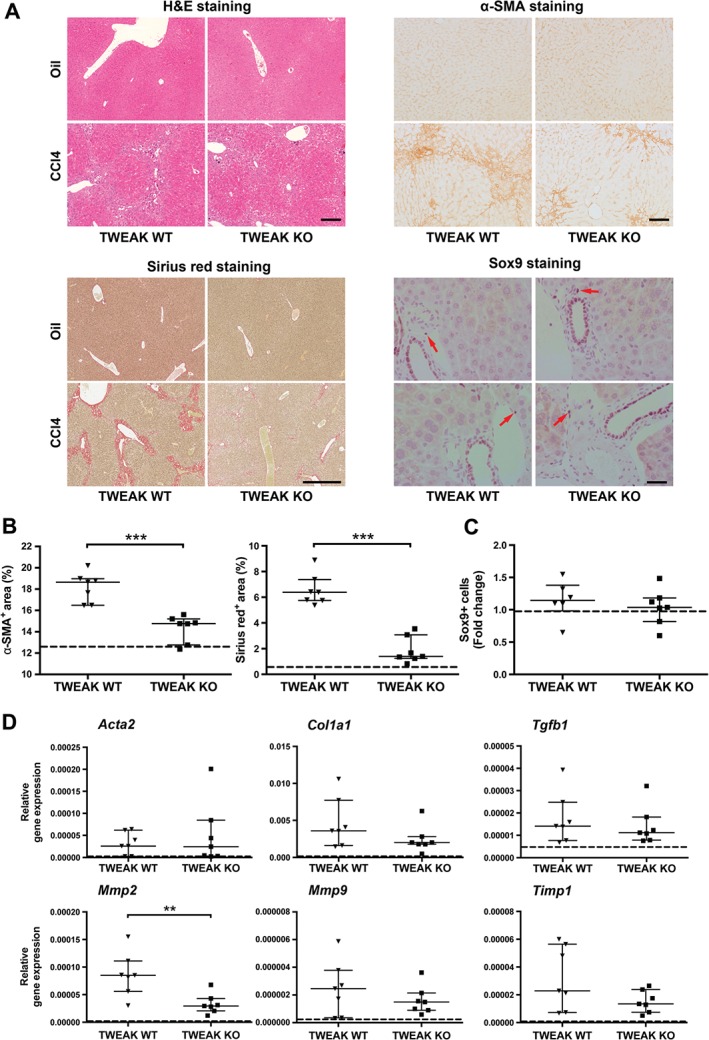
TWEAK KO mice present with reduced liver fibrosis upon chronic CCl_4_ treatment: TWEAK KO and WT mice were dosed with CCl_4_ once weekly for 4 weeks, then killed 3 days after the final dose; Mice treated with mineral oil were used as a control. (A) Representative images of liver tissue sections stained with H&E (scale bar = 200 µm), α‐SMA antibody (scale bar = 100 µm), Sirius red (scale bar = 600 µm) and Sox9 (scale bar = 50 µm); red arrows, liver progenitor cells. (B) Digital quantification of α‐SMA and Sirius red‐positive areas, expressed as percentage area of 10 randomly selected areas/sample or of one whole liver section, respectively (n = 5–7). (C) Average number of Sox9‐positive cells of 10 perivascular areas/sample, presented as fold change to mineral oil treated controls (n = 5–7). (D) Levels of fibrosis‐associated transcripts were analysed by qPCR in chronic CCl_4_‐treated TWEAK KO mice and TWEAK WT mice (n = 7); expression is shown relative to Rn18s, using the 2^–ΔCt^ method; data are shown as median with IQR; each dot represents one mouse; statistical significance is represented as **p < 0.01 or ***p < 0.005 (Mann–Whitney U‐test); dashed line, mRNA levels of mineral oil control‐treated TWEAK KO and WT mice

To examine the effect of TWEAK on chronic liver fibrosis, TWEAK KO and TWEAK WT mice were treated weekly with CCl_4_ for 4 weeks and the extents of liver injury and fibrosis were analysed. Histological examination of livers from TWEAK KO and WT mice treated with CCl_4_ chronically revealed foci of centrilobular necrosis and inflammation in addition to bridging necrosis between the central veins. The hepatocytes around the portal tracts were unaffected. Following CCl_4_ treatment, TWEAK KO mice exhibited reduced areas of α‐SMA and Sirius red staining in the liver compared to WT control animals (Figure 6A). α‐SMA^+^ cells displayed a zonal distribution and Sirius red^+^ collagen was present in fibrous septa and portal areas in mice with CCl_4_ injury. Image analysis revealed significantly fewer myofibroblasts (α‐SMA^+^) and reduced collagen deposition (Sirius red staining) in TWEAK KO animals compared to TWEAK WT mice (Figure 6B); however, no difference in the number of LPCs and ductular cells was found, as indicated by the same number of Sox9^+^ cells in TWEAK KO and WT mice following CCl_4_ treatment (Figure 6C). In addition, TWEAK KO mice had reduced *Mmp2* mRNA levels compared to their WT controls, similar to that seen for the acute model (Figure 6D). The abundance of *Acta2*, *Col1a1*, *Tgfb1*, *Mmp9* and *Timp1* remained unchanged (Figure 6D).

## Discussion

Here we have provided evidence for a pivotal role for TWEAK in the progression of fibrotic liver disease. Fn14 and TWEAK expression were elevated in chronic end‐stage human liver disease and correlated positively with the extent of fibrosis. Localization of TWEAK and Fn14, as revealed by immunohistochemical methods, demonstrated that liver myofibroblasts can express both the ligand and the receptor. Furthermore, we demonstrated expression of Fn14 in quiescent and activated primary human HSCs and showed that exogenous TWEAK induced HSCs proliferation, consistent with a role in the development of liver fibrosis. The role of TWEAK as a novel stimulator of liver fibrogenesis *in vivo* was evidenced by reduced fibrosis in TWEAK KO animals subjected to acute and chronic CCl_4_‐induced liver injury.

Fn14 expression has mainly been associated with LPCs and biliary epithelial cells 9, 10, 12. The liver diseases PSC, PBC and AIH are commonly associated with aberrant ductular regeneration and, consistent with this, we found increased Fn14 (*TNFRSF12A*) mRNA and protein levels and expression of Fn14 associated with CK19^+^ ductule‐like structures in end‐stage liver disease explants. Furthermore, a significant up‐regulation of Fn14 protein was observed in tissue sections taken from patients with ALD and NASH, which revealed the co‐expression of Fn14 with α‐SMA^+^ myofibroblasts in the fibrotic scar. The only known ligand for Fn14 thus far, TWEAK, has been identified in natural killer cells and macrophages in the liver 9, although to our knowledge the pattern of TWEAK expression in human liver tissue has not been studied in depth. We found that TWEAK was up‐regulated in chronic liver diseases, and was associated not only with infiltrating leukocytes but also with HSCs and myofibroblasts. This suggests that myofibroblasts can express TWEAK or/and Fn14, which raises the possibility that TWEAK may exert its function on HSCs in a paracrine/autocrine manner during liver injury. Previously published data by Hotta *et al* 20 support this hypothesis in a model of acute kidney injury, where TWEAK produced by tubules acted via an autocrine loop through up‐regulation of the Fn14 receptor. Furthermore, it has previously been suggested that Kupffer cells might express TWEAK 12 and that depletion of Kupffer cells with clodronate *in vivo* leads to a reduction in TWEAK expression in the liver 21. Therefore, Kupffer cells are likely to be another source of TWEAK within the inflammatory micro‐environment during liver disease.

It is thought that the principal function of TWEAK is to induce ductal proliferation and LPCs expansion mediated through Fn14 8, 11, 12. However, we have demonstrated that HSCs can also express Fn14 and maintain expression during spontaneous activation on plastic *in vitro*. Prior to HSCs activation, as indicated by the detection of α‐SMA on day 14 (Figure 4A), the staining pattern for Fn14 was perinuclear and thus suggestive of Golgi/ER accumulation, whereas Fn14 expression became more diffuse and cytoplasmic following HSCs activation. Our data are in accordance with a previous demonstration that Fn14 is present in the Golgi apparatus and that it can be quickly trafficked to and from the cell surface, undergoing continuous *de novo* receptor synthesis 22. Our data demonstrated that Fn14 expression in HSCs was present prior to the expression of the activation marker α‐SMA, suggesting that Fn14 is regulated by transcription factors that play a role in early cellular transformation and that Fn14 expression might be induced during the early stages of fibrosis. Enhanced Fn14 expression during cell transformation has also been demonstrated in the corneal stroma, where quiescent keratocytes start expressing Fn14 after injury 23. As Fn14 expression was enhanced in HSCs during phenotypic transdifferentiation, we investigated factors that could augment Fn14 expression. Therefore, HSCs were treated with cytokines known to induce Fn14 expression in other cell types 24, in addition to those that have been detected in fibrotic liver tissue, such as bFGF, TNFα, IFNγ and TGFβ 25. Stimulation of HSCs by TGFβ1 led to a significant up‐regulation of *Fn14* mRNA and protein cell surface expression in activated HSCs. The pro‐inflammatory cytokines TNFα and IFNγ were less effective in modulating Fn14 expression, suggesting that pro‐fibrogenic cytokines, rather than pro‐inflammatory factors, induce Fn14 expression and promote cell surface expression in HSCs.

The immunostaining of liver sections showed that TWEAK can be expressed by myofibroblasts and, consistent with these results, activated HSCs secreted low levels of TWEAK into their culture medium, providing further support for an Fn14–TWEAK autocrine/paracrine loop. The co‐expression of TWEAK and Fn14 has also been detected on other cell types, including primary proximal tubular epithelial cells and human blastoma cell lines 20, 26.

During fibrogenesis, HSCs differentiate, proliferate and produce ECM. We therefore investigated how TWEAK might affect HSCs function. Our data show that TWEAK acts as a novel mitogen for human HSCs but, intriguingly, the expression of *ACTA2* (α‐SMA) decreased following TWEAK stimulation. This has also been demonstrated in keratocytes, where TWEAK inhibited transformation into myofibroblasts *in vitro* 23. In addition, it has been demonstrated in a number of other cell types that TWEAK can regulate proliferation and differentiation. In hepatoblasts, skeletal myoblasts and osteoblasts, TWEAK promoted proliferation but inhibited differentiation 27, 28, 29. Furthermore, our data also demonstrate that TWEAK stimulation had no effect on collagen synthesis in HSCs. In contrast, other studies have demonstrated that TWEAK induces collagen expression in cardiac fibroblasts via Fn14 30, 31. TWEAK might therefore be important in expanding the HSC population, at which point pro‐fibrogenic factors such as TGFβ1 are required to enhance the differentiation and broader fibrotic response.

Insights into the role of TWEAK and Fn14 in liver fibrosis have arisen from experimental models of ductular reaction and liver regeneration 9, 16. We therefore investigated the effect of TWEAK in models of acute and chronic CCl_4_‐induced liver injury model primarily associated with fibrosis. Our data demonstrated that acute and chronic toxic liver damage resulted in a significant up‐regulation of Fn14, whereas *TWEAK* mRNA did not change significantly throughout acute or chronic CCl_4_ injury. These results were similar to previously published data in mice on the CDE diet 9, 14. Our data also showed that *TWEAK* KO mice developed less fibrosis following both acute and chronic CCl_4_ injury, as demonstrated by significant reductions in collagen deposition and myofibroblast activation, clearly demonstrating the role of TWEAK as a stimulator of liver fibrogenesis *in vivo*. As TWEAK has been associated with an expansion of ductular cells and LPCs in other models 9, 11, 12, we also investigated whether CCl_4_‐challenged *TWEAK* KO mice had reduced numbers of Sox9^+^ ductular cells compared to their WT controls. Our data demonstrated that there was no difference in the number of Sox9^+^ cells between KO and WT mice. This further supports our theory that CCl_4_‐induced fibrosis is mediated through the interaction of TWEAK and HSCs and is not solely due to TWEAK‐mediated LPCs proliferation that drives HSCs activation and fibrogenesis.

In conclusion, our findings suggest that TWEAK can regulate the fibrogenic response following liver injury. Furthermore TWEAK can induce the expansion of activated HSCs through an enhanced proliferative response. Therapies aimed at modulation of TWEAK expression could therefore be effective modalities for amelioration of fibrogenic responses during liver injury.

## Author contributions

AW, DHA, LCB, CJW and SCA were involved in study concept and design; AW, ELS, AA, GR and CJW acquired the data; AW, ELS, YR, SH, LCB, CJW and SCA analysed and interpreted the data; AW prepared the manuscript; AW, AA, YR, DHA, SH, LCB, CJW and SCA were involved in the drafting of the manuscript; MM, GR and EH provided scientific support; LCB, SCA provided material and laboratory facilities support; and SCA was the project fund holder.

## Abbreviations

AIH, autoimmune hepatitis; ALD, alcoholic liver disease; ALF, acute liver failure; α‐SMA, α‐smooth muscle actin; CCl_4_, carbon tetrachloride; CDE, choline‐deficient, ethionine‐supplemented; ECM, extracellular matrix; Fn14, fibroblast growth factor‐inducible 14; HSCs, hepatic stellate cells; IQR, interquartile range; LPCs, liver progenitor cells; MFI, median fluorescent intensity; NASH, non‐alcoholic steatohepatitis; NL, normal liver; PBC, primary biliary cirrhosis; PSC, primary sclerosing cholangitis; sTWEAK, soluble TWEAK; TNFSF12, tumour necrosis factor ligand superfamily member 12; TNFRSF12A, tumour necrosis factor receptor superfamily member 12A; TWEAK, tumour necrosis factor‐like weak inducer of apoptosis.

**Supplementary material on the internet**

The following supplementary material may be found in the online version of this article:

Supplementary materials and methods

**Figure S1.** Positive correlation between the extent of fibrosis and TWEAK/Fn14 in human liver samples

**Figure S2.** Fn14 is expressed by biliary epithelial cells and ductular reactive cells in human chronic liver disease

**Table S1.** Human PCR primers and probes

**Table S2.** qPCR cycling conditions for human studies

**Table S3.** qPCR cycling conditions for mouse studies

## Supporting information


**Figure S1.** Positive correlation between the extent of fibrosis and TWEAK/Fn14 in human liver samples: extent of fibrosis was measured in normal and cirrhotic livers (NL n = 3, NASH n = 3, ALD n = 2, AIH n = 3) and compared to: (A) Fn14 mRNA; (B) TWEAK mRNA; (C) Fn14 protein; and (D) in normal and cirrhotic livers (NL n = 2, NASH n = 3, AIH n = 1) and compared to TWEAK protein (Spearman rho correlation)

**Figure S2.** Fn14 is expressed by biliary epithelial cells and ductular reactive cells in human chronic liver disease: representative confocal images showing localization of Fn14 (green)‐expressing cells relative to those expressing the biliary marker CK19 (red) in tissue samples from patients with NASH or PSC; co‐localization of Fn14 and CK19 resulted in pseudocolour yellow; (inset) digitally enlarged image; DAPI (blue) was used as a nuclear counterstain; scale bar = 50 µm

**Table S1.** Human primers and probes

**Table 2.** qPCR cycling conditions for human studies

**Table S3.** qPCR cycling conditions for mouse studies

**Appendix S1.** Supplementary materials and methods
