# Supplementary figures and images for: Interaction of TWEAK with Fn14 leads to the progression of fibrotic liver disease by directly modulating hepatic stellate cell proliferation
Source: J Pathol. 2016 Mar 29;239(1):109–21. doi: 10.1002/path.4707 (PMC4949530; doi:10.1002/path.4707)

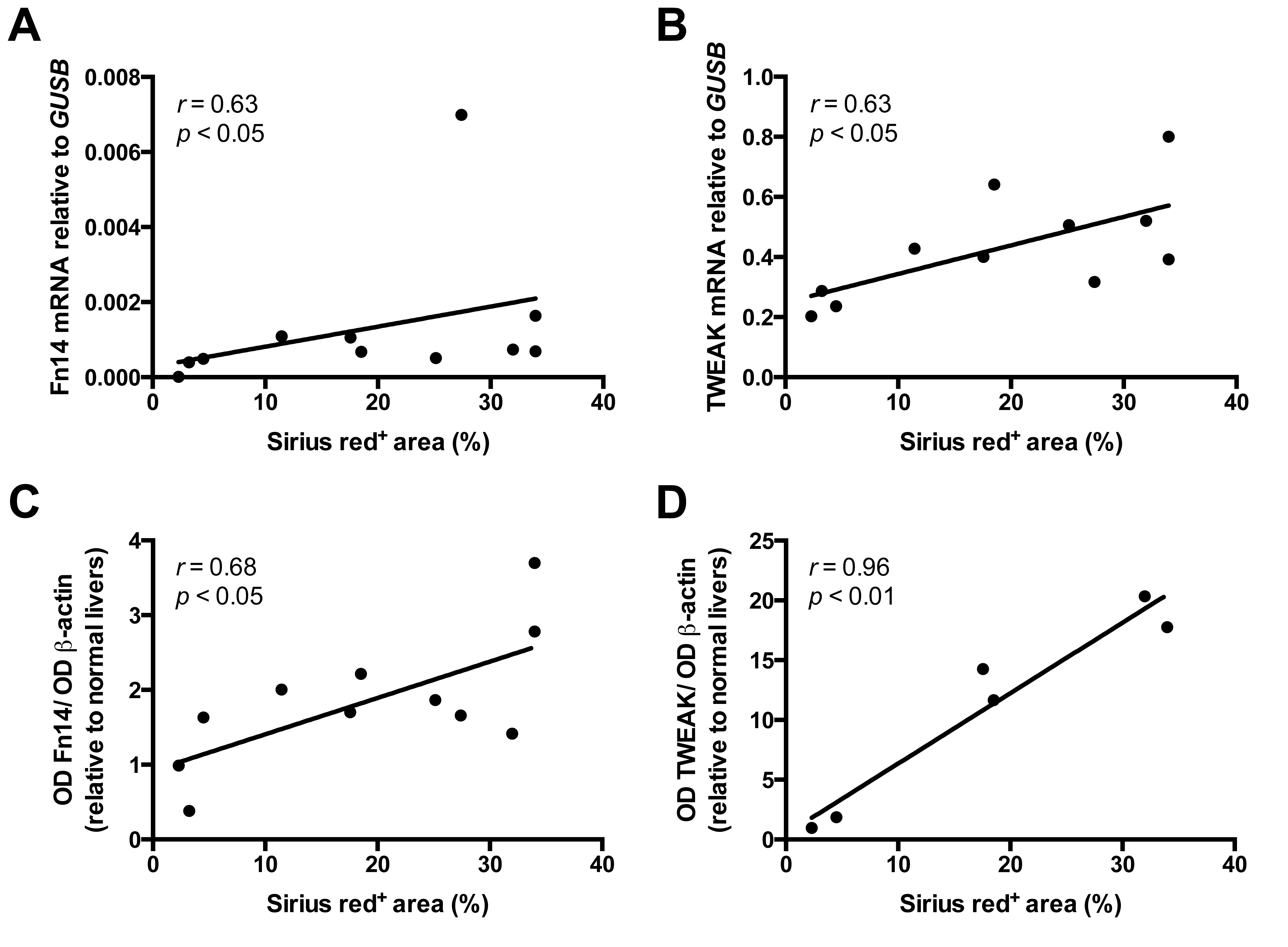

Supplement: Supplementary file 1 — Figure S1. Positive correlation between the extent of fibrosis and TWEAK/Fn14 in human liver samples: extent of fibrosis was measured in normal and cirrhotic livers (NL n = 3, NASH n = 3, ALD n = 2, AIH n = 3) and compared to: (A) Fn14 mRNA; (B) TWEAK mRNA; (C) Fn14 protein; and (D) in normal and cirrhotic livers (NL n = 2, NASH n = 3, AIH n = 1) and compared to TWEAK protein (Spearman rho correlation) [file PATH-239-109-s001.tif]

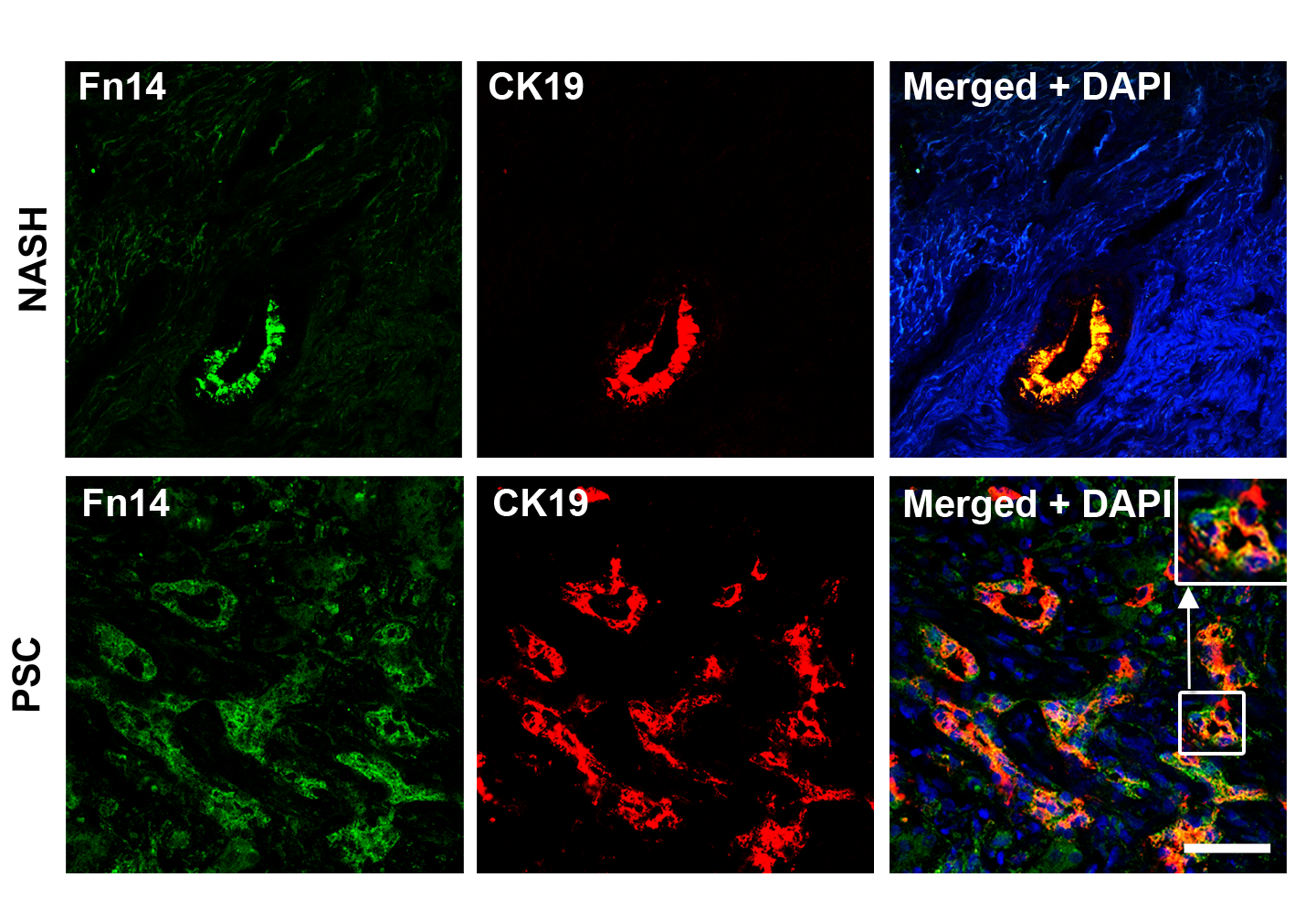

Supplement: Supplementary file 2 — Figure S2. Fn14 is expressed by biliary epithelial cells and ductular reactive cells in human chronic liver disease: representative confocal images showing localization of Fn14 (green)‐expressing cells relative to those expressing the biliary marker CK19 (red) in tissue samples from patients with NASH or PSC; co‐localization of Fn14 and CK19 resulted in pseudocolour yellow; (inset) digitally enlarged image; DAPI (blue) was used as a nuclear counterstain; scale bar = 50 µm [file PATH-239-109-s002.tif]
