# Supplementary material for: Interaction of TWEAK with Fn14 leads to the progression of fibrotic liver disease by directly modulating hepatic stellate cell proliferation
Source: J Pathol. 2016 Mar 29;239(1):109–21. doi: 10.1002/path.4707 (PMC4949530; doi:10.1002/path.4707)
Supplement: Supplementary file 3 — Table S1. Human primers and probes [file PATH-239-109-s003.docx]

**Table S1.** Human primers and probes

| **Target** | **Forward primer (5′→3′)** | **Reverse primer (5′→3′)** | **Probe^#^ (Roche)** |
| --- | --- | --- | --- |
| *TNFRSF12A* | GACCGCACAGCGACTTCT | CACGAAGGTCAGGCTCAGA | 26 |
| *TNFSF12* | ATCGCAGCCCATTATGAAGT | CTCACTGTCCCGTCCACAC | 85 |
| *ACTA2* | CTGTTCCAGCCATCCTTCAT | TCATGATGCTGTTGTAGGTGGT | 58 |
| *COL1A1* | GGGATTCCCTGGACCTAAAG | GGAACACCTCGCTCTCCA | 67 |
|  |  |  |  |
| Reference gene | |  |  |
| *GUSB* | Roche cat. no. 05190525001 |  |  |
