# Supplementary material for: Interaction of TWEAK with Fn14 leads to the progression of fibrotic liver disease by directly modulating hepatic stellate cell proliferation
Source: J Pathol. 2016 Mar 29;239(1):109–21. doi: 10.1002/path.4707 (PMC4949530; doi:10.1002/path.4707)
Supplement: Supplementary file 4 — Table 2. qPCR cycling conditions for human studies [file PATH-239-109-s004.docx]

**Table 2.** qPCR cycling conditions for human studies

| **Cycle** | **Temperature**  **(°C)** | **Time**  **(min:s)** |  |
| --- | --- | --- | --- |
| Pre-incubation | 95 | 10:00 |  |
| Amplification | 95 | 00:10 | Cycling ×45 |
|  | 60 | 00:30 |  |
|  | 72 | 00:01 |  |
| Cooling | 40 | 00:10 |  |
