# Supplementary material for: Interaction of TWEAK with Fn14 leads to the progression of fibrotic liver disease by directly modulating hepatic stellate cell proliferation
Source: J Pathol. 2016 Mar 29;239(1):109–21. doi: 10.1002/path.4707 (PMC4949530; doi:10.1002/path.4707)
Supplement: Supplementary file 5 — Table S3. qPCR cycling conditions for mouse studies [file PATH-239-109-s005.docx]

**Table S3.** qPCR cycling conditions for mouse studies

| **Cycle** | **Temperature**  **(°C)** | **Time**  **(min:s)** |  |
| --- | --- | --- | --- |
| Pre-incubation | 95 | 02:00 |  |
| Amplification | 95 | 00:15 | Cycling ×50 |
|  | 60 | 01:00 |  |
| Cooling | 40 | 00:10 |  |
