# Supplementary material for: Interaction of TWEAK with Fn14 leads to the progression of fibrotic liver disease by directly modulating hepatic stellate cell proliferation
Source: J Pathol. 2016 Mar 29;239(1):109–21. doi: 10.1002/path.4707 (PMC4949530; doi:10.1002/path.4707)
Supplement: Supplementary file 6 — Appendix S1. Supplementary materials and methods [file PATH-239-109-s006.docx]

+A: **Supplementary materials and methods**

+B: Quantitative PCR analysis

Total RNA was extracted using the Qiagen RNeasy Mini Kit (Qiagen, CA, USA) according to the manufacturer’s instructions. Complementary DNA was synthesized from 1 or 2 μg human or mouse RNA, using an iScript cDNA Synthesis Kit (Biorad, UK) or Precision-RT-Premix (Primerdesign, Southampton, UK), respectively. For human studies, cDNA was amplified using Lightcycler 480 probes (Roche, UK) and primers (Alta Biosciences, UK; see Table S1) with the settings outlined in Table S2. Primers for β-glucuronidase (*GUSB*; Roche) served as an internal reference. For mouse studies, Taqman gene expression assays were used: *Tnfrsf12a* (Fn14, Mm01302476_g1); *Tnfsf12* (TWEAK, Mm02583406_s1); *Acta2* (Mm00808218_g1); *Col1a1* (Mm00801666_g1); *Timp1* (Mm00441818_m1); *Mmp2* (Mm00439498_m1); *Mmp9* (Mm00442991_m1); *Tgfb1* (Mm01178820_m1). Levels of the target gene transcripts were normalized to a housekeeping gene, *Rn18s* (Mm03928990_g1) or *Gapdh* (Mm99999915_g1), after evaluating six commonly used housekeeping genes using the geNorm reference gene selection kit (Primerdesign). Assays were performed in a LightCycler 480 System (Roche) with the settings outlined in Table S3. RNA quantity and quality were assessed with a Nanophotometer (Implen Geneflow); an OD_260/280_ value of 1.8–2.0 was deemed acceptable; gene expression values are stated as 2^–^*^ΔC^*^t^.

+B: Western blotting

Liver tissues were lysed with CelLytic MT buffer (Sigma) supplemented with proteinase inhibitor (Roche) and DNaseI (Sigma), using a tissue homogenizer. Proteins (50–60 μg/lane) were separated on a 15% SDS polyacrylamide gel, blotted onto Hybond ECL membrane (GE Healthcare) and immunoblotted with primary antibodies: Fn14 (1:1000; New England Biolabs, Hertfordshire, UK), TWEAK (1:100; 3.24 μg/ml, mP2D10, Biogen) and β-actin (1:15000; Sigma) followed by horseradish peroxidase-conjugated secondary antibody. Detection of the bound antibody was performed using enhanced chemiluminescence western blotting substrate (Thermo Scientific). ImageJ software v. 1.64 was used to analyse western blot images.

+B: Flow cytometry of HSCs

HSCs were trypsinized, then washed with 2% FCS/PBS/1 mm EDTA. Cell suspensions were then incubated with unconjugated mouse anti-Fn14 (3.7 μg/ml; mP4A8) and labelled with PE-conjugated anti mouse secondary (BD Bioscience). Control samples were labelled with isotype-matched control antibodies (Life Technologies). Cells were analysed on a Cyan ADP analyser (Dako) and FlowJo software v. 8.7.

+B: TWEAK ELISA

Soluble TWEAK (sTWEAK) levels were quantified in tissue culture supernatant from HSCs, using human TWEAK instant ELISA (eBioscience, Hatfield, UK) according to the manufacturer’s instructions. In brief, HSCs were seeded at 30 000 cells/well of a 24-well plate, then serum-starved in DMEM containing 0.5% BSA (Sigma) for 24 h. The medium was then substituted for DMEM containing 16% fetal calf serum (FCS) and the conditioned medium was harvested after a further 24 h. Each sample was run in duplicate and the sTWEAK concentration was determined by comparison to the standard concentration curve; values are expressed as pg/ml; the sensitivity of the assay was 9.7 pg/ml.
